# The essential role of fructose-1,6-bisphosphatase 2 enzyme in thermal homeostasis upon cold stress

**DOI:** 10.1038/s12276-020-0402-4

**Published:** 2020-03-16

**Authors:** Hyun-Jun Park, Hye Rim Jang, Shi-Young Park, Young-Bum Kim, Hui-Young Lee, Cheol Soo Choi

**Affiliations:** 1 Laboratory of Mitochondrial and Metabolic Diseases, Department of Health Sciences and Technology, GAIHST, Gachon University, Incheon, 21999 Korea; 2 Department of Medicine, Gachon University College of Medicine, Incheon, 21565 Korea; 3 Korea Mouse Metabolic Phenotyping Center, Lee Gil Ya Cancer and Diabetes Institute, Gachon University, Incheon, 21999 Korea; 4 Division of Endocrinology, Diabetes and Metabolism, Department of Medicine, Beth Israel Deaconess Medical Center, Harvard Medical School, Boston, MA 02215 United States; 5 Division of Endocrinology and Metabolism, Department of Internal Medicine, Gil Medical Center, Gachon University College of Medicine, Incheon, 21565 Korea

**Keywords:** Homeostasis, Metabolic syndrome

## Abstract

Skeletal muscle is a major organ for glucose disposal and thermogenesis. While hepatic fructose-1,6-bisphosphatase is well known as a key enzyme for gluconeogenesis, the role of muscle fructose-1,6-bisphosphatase 2 (Fbp2) in glucose disposal and thermogenesis is unknown. Here, using Fbp2 knockout (KO) mice, we assessed the physiological role of Fbp2 in energy and glucose metabolism and thermogenesis. In vivo assessments of energy metabolism, glucose metabolism, and thermogenesis were performed by indirect calorimetry, hyperinsulinemic-euglycemic clamp, and cold challenge studies, respectively. Under both feeding and fasting conditions, Fbp2 KO mice showed similar phenotypes regarding energy and glucose metabolism compared to wild-type (WT) mice. However, Fbp2 KO mice were severely intolerant to cold challenge under fasting conditions. Mechanistically, the cold-induced intramuscular conversion of lactate to glycogen (glyconeogenesis) is completely abolished in the KO muscle, which leads to a lack of glycogen source for thermogenesis in Fbp2 KO mice. The cold-intolerant phenotype of KO mice disappeared after feeding, and the KO mice were equally as cold tolerant as the WT mice and survived during the cold challenge for three weeks. Taken together, these data demonstrate that Fbp2 is essential for muscle thermogenesis by replenishing the intramuscular glycogen pool through glyconeogenesis when the exogenous glucose source is limited. These data imply the physiological importance of Fbp2 in thermal homeostasis and suggest a potential novel therapy targeted to increase glycogen replenishment upon cold stress.

## Body temperature: Enzyme critical to heat production in muscle

When simple sugars in the diet are scarce, skeletal muscle can still generate heat under cold conditions thanks to an enzyme that converts a metabolic byproduct into complex carbohydrates. A team led by Hui-Young Lee and Cheol Soo Choi from Gachon University’s Lee Gil Ya Cancer and Diabetes Institute in Incheon, South Korea, showed that, under fasting conditions, mice lacking a muscle form of enzyme called fructose-1,6-bisphosphatase 2 (Fbp2) could not respond to cold exposure by the usual process of converting lactate, which builds up in muscles during intense activity, into glycogen, a type of complex sugar involved in heat production not related to shivering. After a meal, however, the same mice could adapt to extreme cold without any problem. The findings highlight the importance of Fbp2 in thermal regulation under fasting conditions.

## Introduction

Skeletal muscle is a major glucose-consuming organ that occupies ~40% of body mass^1^, and its insulin resistance is a primary defect in pandemic metabolic syndrome and type 2 diabetes worldwide^2^. A large body of research has been focused on muscle contraction, growth, and exercise performance due to its relevance to health and metabolic disorders^3^; however, these studies have placed less emphasis on the role of skeletal muscle in regulating body temperature and metabolism. Generally, it has been assumed that heat production and metabolic activity in skeletal muscle are primarily caused by contractile activity because muscle shivering is recruited as the first line of defense during acute exposure to cold^4^. Shivering is a repetitive contraction-relaxation process that is activated by repeated stimulation of the neuromuscular junction that leads to an elevation of cytosolic Ca^2+^ concentration, thereby activating adenosine triphosphate (ATP) hydrolysis to produce heat^3^. During shivering, heat is primarily produced by the major ATP-utilizing enzymes, including Na^+^/K^+^ ATPase, myosin ATPase, and sarco/endoplasmic reticulum Ca^2+^-ATPase^5,6^. Lipids provide most of the heat during low-intensity shivering, whereas carbohydrates become dominant under more extreme cold conditions^7^. The contribution from plasma glucose always remains minor; however, muscle glycogen plays an important role during intense shivering^7^. Although acute increases in exogenous glucose uptake and glycogen synthesis by insulin primarily maintain muscle glycogen size^8^, the glycogen pool can be replenished from 3-carbon units, such as lactate, named glyconeogenesis^9,10^. However, whether the size of the muscle glycogen stores influences cold tolerance as well as the physiological importance of glyconeogenesis remain to be demonstrated.

In mammals, there are liver and muscle isotypes of fructose-1,6-bisphosphatase (Fbp), encoded by two separate genes, Fbp1 and Fbp2. The Fbp1 gene is mainly expressed in gluconeogenic organs, such as the liver and kidneys, and is the regulatory enzyme of gluconeogenesis^11^. Fbp1 plays a key role in hepatic gluconeogenesis. In animal models, the inhibition of Fbp1 markedly inhibits gluconeogenesis and increases glucose sensitivity and utilization^12^. In humans, Fbp1 deficiency caused by Fbp1 mutations decreases gluconeogenesis^13^ and leads to hypoglycemia, metabolic acidosis during fasting^14,15^, and unexpected infant death^16^. In contrast, the Fbp2 gene is expressed in nongluconeogenic organs and was identified in striated muscle by Krebs and Woodford in 1965^17^. In skeletal muscle, Fbp2 catalyzes the conversion of fructose-1,6-bisphosphate into fructose-6-phosphate and inorganic phosphate^18^ and is located in the middle of the glyconeogenesis and glycolytic pathways. Several studies have attempted to clarify the physiological meaning of Fbp2, which is distinguished from the gluconeogenic isotype Fbp1. A hypothesis was proposed by Newsholme in 1976 that the futile cycle between fructose-6-phosphate and fructose-1,6-bisphosphate mediated by Fbp2 could regulate whole-body thermal homeostasis^18^. Furthermore, the activity of Fbp2 has been known to be increased by cold challenge^19^ as well as translocated to the mitochondria by glycogen synthase kinase 3 inhibition^20,21^, suggesting its role in thermogenesis. However, whether Fbp2 directly contributes to muscle thermogenesis in vivo and the underlying mechanisms have not been investigated. A study using Fbp2 knockout (KO) animals has not been reported, and there is limited clinical evidence that Fbp2 deficiency is associated with benign nonprogressive myopathy in humans^22^. Thus, it is a testable hypothesis as to whether Fbp2 may affect intramuscular glycolysis, glyconeogenesis, or mitochondrial respiration, each of which could contribute to energy metabolism and thermogenesis. Here, we performed comprehensive studies using Fbp2 KO and littermate control (wild-type: WT) mice to identify the role of Fbp2 in energy and glucose metabolism and thermal homeostasis in vivo.

## Materials and methods

### Mice, body composition, and energy metabolism

Fbp2 knockout (KO) mice were kindly provided by Dr. Dong Kong and Dr. Brad Lowell (Division of Endocrinology, Department of Medicine of Beth Israel Deaconess Medical Center and Harvard Medical School, Boston, MA. Please contact dong.kong@tufts.edu or blowell@bidmc.harvard.edu for details regarding the generation of the mouse model). The Fbp2 KO mice were backcrossed with the C57BL/6J strain over five generations before being used in this study in the animal facilities of the Lee Gil Ya Cancer and Diabetes Institute (Gachon University, Incheon, Korea). Animals were housed under controlled room temperature (22 ± 2 °C) and a 12-h light/dark cycle with free access to water and food ad libitum with a regular chow diet (5053, Labdiet, St. Louis, MO, USA) or a high-fat diet (D12492, Research Diets, New Brunswick, NJ, USA).

The fat and lean masses of mice were analyzed by an ^1^H-nuclear magnetic resonance system (Bruker Optics, Billerica, MA, USA) and expressed as grams. To assess whole-body energy metabolism, a comprehensive laboratory animal monitoring system (CLAMS, Columbus Instruments, Columbus, OH, USA) was applied. The oxygen consumption rate (VO_2_), carbon dioxide production (VCO_2_), respiratory quotient, energy expenditure, food intake and activity were monitored for 72 h after a 24-h acclimation period. All experimental protocols were approved by the Institutional Animal Care and Use Committee of Lee Gil Ya Cancer and Diabetes Institute, Gachon University (Permission number: 2017-0055).

### Hyperinsulinemic-euglycemic clamp

Mice were maintained on a regular chow diet or high-fat diet (60 kcal%) for 4 weeks. Six to seven days before the hyperinsulinemic-euglycemic clamp, indwelling catheters beneath the back of the neck were positioned in the right internal jugular vein, which extends to the right atrium. To evaluate the basal turnover rate of glucose, [3-^3^H]-glucose (PerkinElmer, Waltham, MA, USA) was infused for 2 h at a rate of 0.05 μCi/min after overnight fasting. After that, a hyperinsulinemic-euglycemic clamp was performed for 150 min with a continuous infusion of human insulin (Eli Lilly and Company, Indianapolis, IN, USA). In detail, insulin was infused at a rate of 3 mU/kg/min for continuous infusion. In order to estimate whole-body glucose fluxes under insulin stimulation, [3-^3^H]-glucose was infused at a rate of 0.1 μCi/min during the clamps. Peripheral glucose uptake and metabolism under insulin-stimulated conditions were estimated by a bolus injection of 2-deoxy-D-[1-^14^C]-glucose (2-DOG) (PerkinElmer, Waltham, MA, USA) at 125 min during the hyperinsulinemic-euglycemic clamp. Whole-body glucose fluxes and glucose uptake of tissues were calculated as previously described^23–25^.

### Cold stress experiments

Individually housed mice aged 11–12 weeks were placed in an environmental chamber (Dae Han Biolink, Eumseong, Korea) after 2 h of fasting. Cold stress experiments of mice at 4 °C were performed for up to 10 h without food but with free access to water. For the long-term cold stress experiment, mice were placed in an environmental chamber with free access to water and food ad libitum. The core temperature of the mice was measured as previously described^26^. To obtain changes in the body temperature of mice, the rectal temperature of mice was measured by using a thermometer coupled with a probe (925, Testo, Lenzkirch, Germany). A 50% (v/v) glycerol solution was used as a lubricant for the thermometer probe to prevent anal damage.

### Immunoblotting

Snap frozen tissues from mouse hindlimb muscle were used for protein extraction. Once tissues were homogenized in liquid nitrogen, tissue lysis buffer (9803S, Cell Signaling Technology, Danvers, MA, USA) supplemented with protease inhibitor cocktail (P8340, Sigma-Aldrich, St. Louis, MO, USA) and phosphatase inhibitor cocktail (P5726, Sigma-Aldrich, St. Louis, MO, USA) was added. Then, lysates were homogenized additionally and incubated for 5 min on ice. Samples were centrifuged for 10 min at 13,200 rpm, and supernatants were used for immunoblotting. Polyacrylamide gel electrophoresis and transfer of proteins to PVDF membranes (IPVH00010, Millipore, Burlington, MA, USA) were conducted for immunoblotting. A 5% (w/v) skim milk solution (232100, Difco, Detroit, MI, USA) based on Tris-buffered saline with 0.1% Tween-20 (IBS-BT007, Intron Biotechnology, Sungnam, Korea) was used for membrane blocking. Antibodies were purchased as below. Fructose-1,6-bisphosphatase 2 (ab131253, Abcam, Cambridge, UK), phospho-PKA substrate (9624S, Cell Signaling Technology, Danvers, MA, USA), uncoupling protein 1 and 3 (ab10983 and ab10985, respectively, Abcam, Cambridge, UK), and goat anti-rabbit IgG antibody (AP132P, Millipore, Burlington, MA, USA) were used.

### RNA isolation and quantitative real-time PCR

Total RNA isolation from cells or tissues was performed by using TRIzol reagent (15596026, Life Technologies, Carlsbad, CA, USA) according to the manufacturer’s instructions. Isolated RNA was subsequently reverse-transcribed to complementary DNA by using the TOPscript^TM^ RT DryMIX kit (RT200, Enzynomics, Daejeon, Korea). Analysis of quantitative real-time PCR was conducted with an ABI 7300 (Applied Biosystems, Foster City, CA, USA) by using TOPreal^TM^ qPCR 2X PreMIX (RT501M, Enzynomics, Daejeon, Korea). The relative mRNA expression of each target was normalized to cyclophilin A or Gapdh. Mouse primer sequences are available on request (CycloA, Gapdh, Fbp1, Fbp2, Pygm, G6pi, Pfk1, Pgm2, Gys1, PGC-1α, Tfam, Nrf-1, Cox5b, Atp5b, Ndufv5).

### Glycogen measurement

The snap frozen hindlimb muscle tissues from mice in cold stress experiments were used for quantitative glycogen content analysis. Tissue samples were homogenized with 0.9N perchloric acid (244252, Sigma-Aldrich, St. Louis, MO, USA) in a mixer at 2000 rpm for 30 s. Then, 50 μL of perchloric acid homogenate was mixed thoroughly with 25 µL of 1M potassium bicarbonate (237205, Sigma-Aldrich, St. Louis, MO, USA). Fresh 4 mg/mL amyloglucosidase (10115, Sigma-Aldrich, St. Louis, MO, USA) in acetate buffer was added to 125 µL of the homogenate mixture, and the final solution was incubated at 56 °C for 4 h. After that, the whole mixture was centrifuged for 1 min at 13,200 rpm, and the supernatant was used to measure the glucose concentration by using a glucose assay kit (GAGO-20, Sigma-Aldrich, St. Louis, MO, USA). The glycogen content was determined by differences in glucose concentrations before and after amyloglucosidase treatment to exclude free glucose from glycogen in skeletal muscle. For the cellular glycogen content assay, a glycogen colorimetric assay kit (K646-100, Biovision, Milpitas, CA, USA) was used according to the manufacturer’s protocol. In detail, cell pellets were homogenized in distilled water on ice. Homogenates were boiled for 10 min to inactivate enzymes and centrifuged at 18,000 × *g* for 10 min. Finally, the supernatant was used to measure the cellular glycogen content according to the protocol provided by the manufacturer.

### Ex vivo ^14^C-lactate incorporation into glycogen in skeletal muscle

The glyconeogenesis in muscle was measured in muscle tissues using a radioisotope-labeled tracer as previously described with slight modifications^10^. Individually housed mice were placed in an environmental chamber after 2 h of fasting. Basal group mice were maintained at room temperature, and cold stress group mice were subjected to 4 °C for 6 h with free access to water only. First, Krebs-Henseleit bicarbonate (KHB) buffer (pH 7.4) containing 20 mM sodium L-lactate (L7022, Sigma-Aldrich, St. Louis, MO, USA), 0.3 μCi/mL L-^14^C(U)-lactic acid, and sodium salt (NEC599050UC, PerkinElmer, Waltham, MA, USA) was gassed with oxygen for 1 h. During oxygenation of the buffer, extensor digitorum longus (EDL) muscle tissues were isolated rapidly from mice. Next, muscle tissues were incubated in the buffer at 37 °C for 1 h and then washed three times with KHB buffer. Incubated tissues were added to 300 μL of 30% KOH solution and heated at 95 °C for 5 min. To isolate the glycogen pellet, 100 μL of 6% w/v Na_2_SO_4_ solution and 600 μL of ethanol were added, and the total mixture was incubated in ice for precipitation. Then, the total mixture was centrifuged at 16,000 × *g* for 10 min, and the supernatant was discarded. The pellet was washed three times with ethanol (70%, 95%, and 99.9% in sequence) and dissolved in distilled water after complete drying. The radioactivity of dissolved glycogen was measured by a liquid scintillation analyzer (Tri-Carb 3110 TR, PerkinElmer, Waltham, MA, USA).

### Primary muscle cell and mouse embryonic fibroblast culture

Mouse primary myoblast and embryonic fibroblast isolation was conducted as previously described^27,28^ with slight modifications. In detail, hindlimb muscle was isolated from neonatal mice (2–7 days old) and rinsed with cold phosphate-buffered saline (ML008-01, Welgene, Daegu, Korea). Isolated muscle tissue was minced with razor blades on a culture dish, and a 2-mL mixture of 1.5 U/mL collagenase (11088866001, Sigma, St. Louis, MO, USA)/2.4 U/mL dispase (D4693, Sigma, St. Louis, MO, USA)/2.5 mM CaCl_2_ solution (21115, Sigma, St. Louis, MO, USA) per gram of tissue was added for further tissue digestion at 37 °C for 20 min. After the tissue was digested and became a fine slurry, cell culture media based on Ham’s F-10 (LM009-01, Welgene, Daegu, Korea) supplemented with 20% v/v FBS (S001-07, Welgene, Daegu, Korea) and 25 μg/mL basic fibroblast growth factor (2.5 ng/mL for final concentration, F0291, Sigma-Aldrich, St. Louis, MO, USA) were added to inactivate collagenase and dispase activity. Next, large clumps and debris were removed through a cell strainer (352350, Falcon, Corning, NY, USA). The whole mixture was centrifuged for 5 min at 350 × *g*, and the pellet was resuspended in cell culture media. Finally, myoblasts were (sub)cultured in type 1 collagen-coated dishes (39006, 21100, 21150, SPL, Pochon, Korea) until a sufficient number of cells was reached. Ham’s F-10 (LM009-01, Welgene, Daegu, Korea) and DMEM/Ham’s F-10 (LM002-112, Welgene, Daegu, Korea) media supplemented with 20% v/v FBS (S001-07, Welgene, Daegu, Korea), 25 μg/mL basic fibroblast growth factor (2.5 ng/mL final concentration, F0291, Sigma-Aldrich, St. Louis, MO, USA) and 1% v/v penicillin/streptomycin (LS202-02, Welgene, Daegu, Korea) were used for myoblast culture. Mouse embryonic fibroblasts were isolated from mouse embryos (E13.5-15.5) and maintained in DMEM supplemented with 10% v/v FBS and 1% v/v penicillin/streptomycin. For the in vitro cold stress-mimicking study, 1 μM isoproterenol (I5627, Sigma-Aldrich, St. Louis, MO, USA) was treated for 4 h in serum-free conditions maintained in low-glucose DMEM (LM001-11, Welgene, Daegu, Korea).

### Cellular and isolated mitochondrial respiration

Cellular and isolated mitochondrial respiration was assessed by an XF24 analyzer (Seahorse Bioscience, Billerica, MA, USA) as previously described with slight modifications^23^. Briefly, the 25 mM glucose-induced oxygen consumption rate was measured in XF assay media (25 mM glucose, 1 mM sodium pyruvate in XF basal media) for cellular respiration analysis. Every reagent was diluted in XF assay media and loaded into ports of the flux plate (1 μg/mL oligomycin, 1 μM carbonyl cyanide p-trifluoromethoxyphenylhydrazone, and 2 μM antimycin A in sequence as the final working concentrations). For isolated mitochondrial respiration analysis, mitochondria were isolated as previously described with slight modifications^25^. In brief, hindlimb muscle was rapidly removed from overnight-fasted mice and homogenized with a dounce homogenizer in isolation buffer (215 mM mannitol, 75 mM sucrose, 0.1% BSA, 1 mM EGTA, 20 mM HEPES, pH 7.2). Then, the homogenate was centrifuged at 800 × *g* for 10 min. The supernatant was centrifuged again at 10,000 × *g* for 10 min, and the mitochondrial pellet was used for downstream analysis. Mitochondria (10 μg/mL) isolated from skeletal muscle were added to each well with respiration assay buffer (220 mM mannitol, 70 mM sucrose, 10 mM KH2PO4, 5 mM MgCl2, 1 mM EGTA, 25 mM MOPS, 0.2% BSA, pH 7.2) supplemented with substrates (10 mM pyruvate, 2.5 mM, malate, 2.5 mM glutamate, and/or 5 mM succinate). Then, every reagent was diluted in respiration assay buffer and loaded into ports of the flux plate (1 mM ADP, 1 μg/mL oligomycin, 1 μM carbonyl cyanide p-trifluoromethoxyphenylhydrazone, and 2 μM antimycin A in sequence as the final working concentrations).

### Statistics

All values are expressed as the mean ± S.E.M. The statistical significance of the mean value difference was analyzed by two-tailed Student’s *t*-test or ANOVA with Bonferroni post hoc analysis using GraphPad Prism 5.0 (GraphPad Software Inc., San Diego, CA, USA). *P*-values less than 0.05 were considered significant differences.

## Results

### Fbp2 was abundant in white skeletal muscle, and Fbp2 deletion changed energy metabolism

**Fig. 1:**
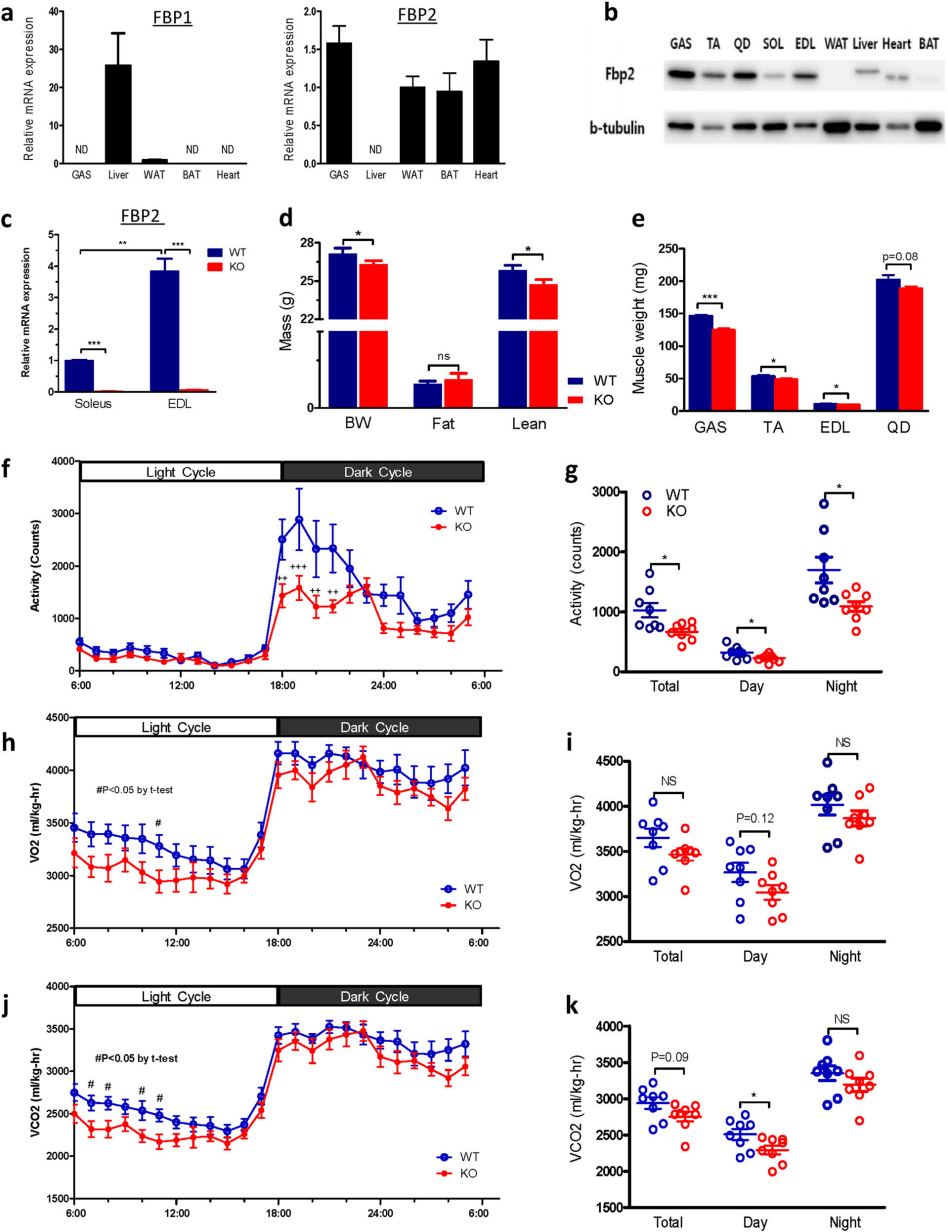
Fbp2 was abundant in white skeletal muscle, and Fbp2 deletion changed energy metabolism. **a** Tissue-specific gene expression patterns of Fbp1 and Fbp2 in WT mice analyzed by quantitative reverse transcription polymerase chain reaction (RT-PCR). ND Nondetected. *n* = 4 per group. **b** Tissue-specific Fbp2 protein expression pattern in WT mice analyzed by immunoblotting. **c** Fbp2 gene expression in the soleus and extensor digitorum longus muscle of WT mice analyzed by quantitative RT-PCR. *n* = 3 per group. **d** Body composition analysis by the ^1^H-nuclear magnetic resonance system including body weight, fat mass, and lean mass. *n* = 8 per group. **e** Weight of various skeletal muscles. *n* = 6 per group. **f** Locomotor activity, **h** whole-body O_2_ consumption (VO_2_), and **j** whole-body CO_2_ production (VCO_2_) of WT and Fbp2 KO mice during the 72-h analysis. *n* = 8 per group. 24 h, day and night time averages of **g** locomotor activity, **i** VO_2_, **k** VCO_2_. GAS gastrocnemius, TA tibialis anterior, QD quadriceps, EDL extensor digitorum longus. Data are expressed as the mean ± SEM. ^*^P < 0.05, ^**^P < 0.01 by Student’s *t*-test for (**c**–**e**, **g**, **i**, **k**). ^++^*P* < 0.01, ^+++^*P* < 0.001 by two-way analysis of variance (ANOVA) with post hoc analysis for (**f**) and ^#^*P* < 0.05 by Student’s t-test at each time point for **h** and **j**. ns nonsignificant.

First, we analyzed the expression patterns of the Fbp2 gene in various tissues from C57BL/6 mice. Although Fbp2 mRNA was broadly expressed in various nongluconeogenic tissues, including white and brown adipose tissues (Fig. 1a), the protein expression of Fbp2 was highly specific to muscle tissues and was not detectable in the liver and adipose tissues (Fig. 1b). In particular, the type 2 fiber abundant extensor digitorum longus (EDL) muscle showed higher Fbp2 gene and protein expression than the type 1 fiber abundant soleus muscle (Fig. 1b, c), which is consistent with previous findings of higher Fbp2 activity in white muscle than in red muscle^29^. Fbp2 gene expression was nondetectable in Fbp2 KO mice (Fig. 1c). Fbp2 KO mice showed slight but significantly lower body weight than WT mice, which was mostly accounted for by decreased lean body mass, as measured by ^1^H-nuclear magnetic resonance (NMR) (Fig. 1d). Indeed, the weight of several skeletal muscles was significantly reduced in KO mice compared to that in WT mice (Fig. 1e), and the decreased muscle mass was associated with reduced locomotor activity during the comprehensive laboratory animal monitoring system study (Fig. 1f, g). Despite the significantly decreased body weight, Fbp2 KO mice showed reductions in oxygen consumption (VO_2_, Fig. 1h, i), carbon dioxide production (VCO_2_, Fig. 1j, k), and energy expenditure (Supplementary Fig. 1b) during the fasting period (light cycle, Fig. 1h, j). The respiratory exchange ratio and food intake were identical between Fbp2 KO and WT mice (Supplementary Fig. 1a, c). There was no difference in energy expenditure at the feeding period between WT and Fbp2 KO mice, inferring a specific role of Fbp2 deletion on energy metabolism when the exogenous fuel supply was limited.

### Fbp2 deletion did not alter glucose metabolism during the feeding condition

**Fig. 2:**
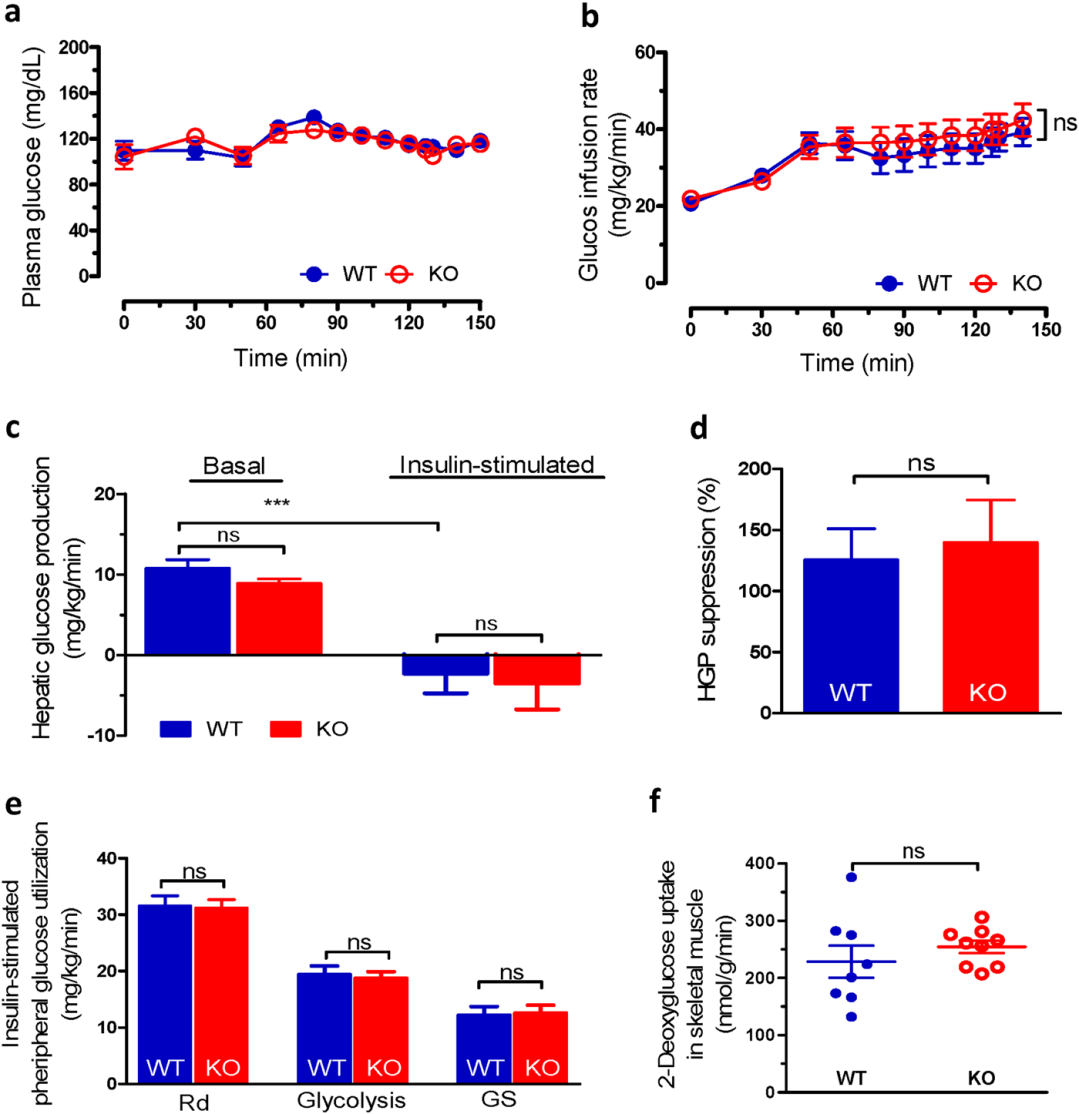
Fbp2 deletion did not alter glucose metabolism during feeding-mimicking conditions. **a** Plasma glucose level, **b** glucose infusion rate during hyperinsulinemic-euglycemic clamp, **c** basal and clamped glucose level, **d** percentage of suppression of hepatic glucose output, and **e** clamped glucose uptake, glycolysis rate, and glycogen synthesis rate calculated using the hyperinsulinemic-euglycemic clamp results. *n* = 9 per group. **f** Muscle 2-DOG uptake results. *n* = 8–9 per group. HGP hepatic glucose production, Rd glucose disposal rate, GS glycogen synthesis rate. Data are expressed as the mean ± SEM and were analyzed by two-way ANOVA with post hoc analysis for **a** and **b** and by Student’s *t*-test for **c**–**g**. ^**^P < 0.01, ^***^P < 0.001, ns nonsignificant.

**Fig. 3:**
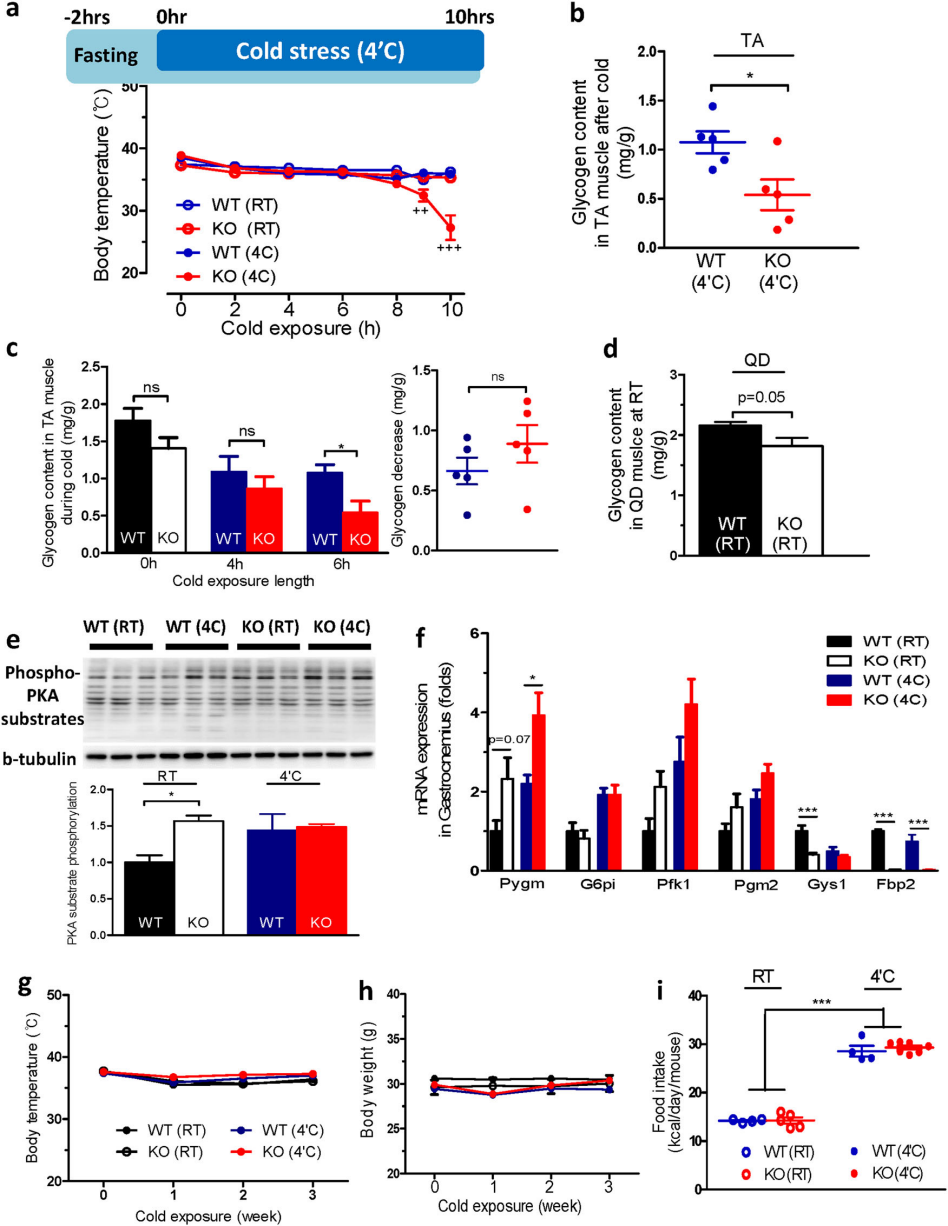
Fbp2 was important for cold tolerance during fasting conditions. **a** Body temperature changes in WT and Fbp2 KO mice in cold stress experiments, *n* = 4 for the RT group; *n* = 8 for the cold group. **b** Glycogen content in the TA and QD muscles of WT and Fbp2 KO mice after cold stress experiments. *n* = 5 per group. **c** Glycogen content depending on cold exposure time (left) and glycogen content decrease after cold stress experiments (right) in the TA muscle of WT and Fbp2 KO mice. *n* = 5 per group. **d** Glycogen content in the QD muscle of WT and Fbp2 KO mice at room temperature. *n* = 5 per group. **e** Immunoblotting results of PKA substrate phosphorylation in WT and Fbp2 KO mice in cold stress experiments. *n* = 5 per group. **f** Gene expression analysis in the GAS muscle of WT and Fbp2 KO mice after cold stress. *n* = 4–6 per group. **g** Body temperature change, **h** body weight change, and **i** daily food intake of WT and Fbp2 KO mice in cold stress experiments for 3 weeks. *n* = 4–7 per group. h hour, TA tibialis anterior, QD quadriceps, PKA protein kinase A, Pygm glycogen phosphorylase, muscle associated, G6pi glucose-6-phosphate isomerase, Pfk1 phosphofructokinase 1, Pgm2 phosphoglucomutase 2, Gys1 glycogen synthase 1. Data are expressed as the mean ± SEM. ^*^P < 0.05, ^**^P < 0.01, ^***^P < 0.001 by Student’s *t*-test. ^++^*P* < 0.01, ^+++^*P* < 0.001 by two-way ANOVA with post hoc analysis for **a**. ns nonsignificant.

Because energy expenditure showed a different pattern between feeding and fasting periods, we further tested whether Fbp2 deficiency alters glucose metabolism in the fed condition. To measure in vivo whole-body and tissue-specific glucose fluxes under feeding-mimicking conditions where insulin and exogenous glucose are supplied, we performed hyperinsulinemic-euglycemic clamp studies on WT and Fbp2 KO mice as previously reported^23,30^. There was no difference in insulin-stimulated whole-body glucose utilization between Fbp2 KO and WT mice, as reflected by the identical glucose infusion rates needed to maintain euglycemia during the steady state (Fig. 2a, b). Hepatic glucose production (HGP) under basal conditions (Fig. 2c) and the suppression of HGP by insulin, which indicates hepatic insulin sensitivity, were not different between Fbp2 KO and WT mice (Fig. 2d). Insulin-stimulated whole-body glucose uptake, glycolysis, and glycogen synthesis rates also showed no differences between WT and Fbp2 KO mice (Fig. 2e). Consistently, skeletal muscle-specific glucose uptake measured by 2-deoxy-D-[1-^14^C]-glucose was identical between WT and Fbp2 KO mice (Fig. 2f). We performed further hyperinsulinemic-euglycemic clamp studies under high-fat-fed conditions. There was no difference in glucose fluxes between WT and KO mice during the clamp study (Supplementary Fig. 2). These data demonstrate that Fbp2 deficiency did not alter glucose metabolism in vivo in either the whole-body or skeletal muscle when glucose was exogenously supplied. However, there was a tendency toward a decrease in glycogen content in various skeletal muscles after fasting (Fig. 3b, c).

### Fbp2 was essential for cold tolerance during fasting conditions

A decreased muscle glycogen content (Fig. 3d) and energy metabolism (Fig. 1) in KO mice were observed only after fasting; therefore, we assumed that Fbp2 might have more crucial roles in the severe glycogenolytic condition. Thus, we further tested the role of Fbp2 in thermogenesis under more severe glycogenolytic conditions by challenging Fbp2 KO mice with both cold and fasting. After 6 h of cold exposure without food, Fbp2 KO mice exhibited more severe cold intolerance than WT mice, and most of the KO mice had to be euthanized after 10 h of cold exposure due to the humane concern of severe hypothermia (Fig. 3a). Fbp2 KO mice showed significantly decreased glycogen content in their tibialis anterior (Fig. 3b) compared to KO mice after cold exposure. We also further tested whether glycogenolysis was accelerated by Fbp2 deletion by measuring the muscle glycogen content at various time points after the cold challenge. Consistent with the decreased body temperature patterns (Fig. 3a), the muscle glycogen content was significantly reduced after 6 h of cold exposure (Fig. 3c) in both WT and KO mice. However, during the 6 h of cold exposure, the net amount of muscle glycogen depletion was identical between WT and KO mice (Fig. 3c, right panel), indicating that cold exposure did not accelerate the glycogenolysis rate by Fbp2 deletion. However, there was a decrease in the basal glycogen content in the quadriceps muscle from overnight-fasted KO mice even at room temperature (22 ± 2 °C) (Fig. 3d). Consistently, phosphorylation of PKA substrates in KO muscle was sufficiently increased compared to that in cold-exposed WT muscle, even at room temperature (Fig. 3e). Furthermore, the mRNA expression of glycogenolytic genes showed an increasing trend in the KO muscle, whereas the glycogen synthase gene was decreased at room temperature (Fig. 3f). These data indicate that the decreased cold tolerance in Fbp2 KO mice is accounted for by the limited fuel source due to the decreased basal content of muscle glycogen but not by an acceleration of glycogenolysis. To further differentiate the effect of exogenous fuel source on body temperature maintenance under cold stress from that of the endogenous form, we conducted a cold challenge experiment with free access to food for longer periods, up to 3 weeks. There was no difference in body temperature (Fig. 3g) or body weight (Fig. 3h) during the 3-week experiment between WT and Fbp2 KO mice. Food intake was equally ~2-fold greater and markedly increased during the cold stress experiment in both WT and KO mice (Fig. 3i). These results demonstrate that Fbp2-mediated thermogenesis did not have a substantial effect on thermal homeostasis when food was supplied but impaired whole-body thermal homeostasis when the exogenous fuel source was limited.

### Fbp2 was necessary for intramuscular glyconeogenesis and affected mitochondrial respiration

**Fig. 4:**
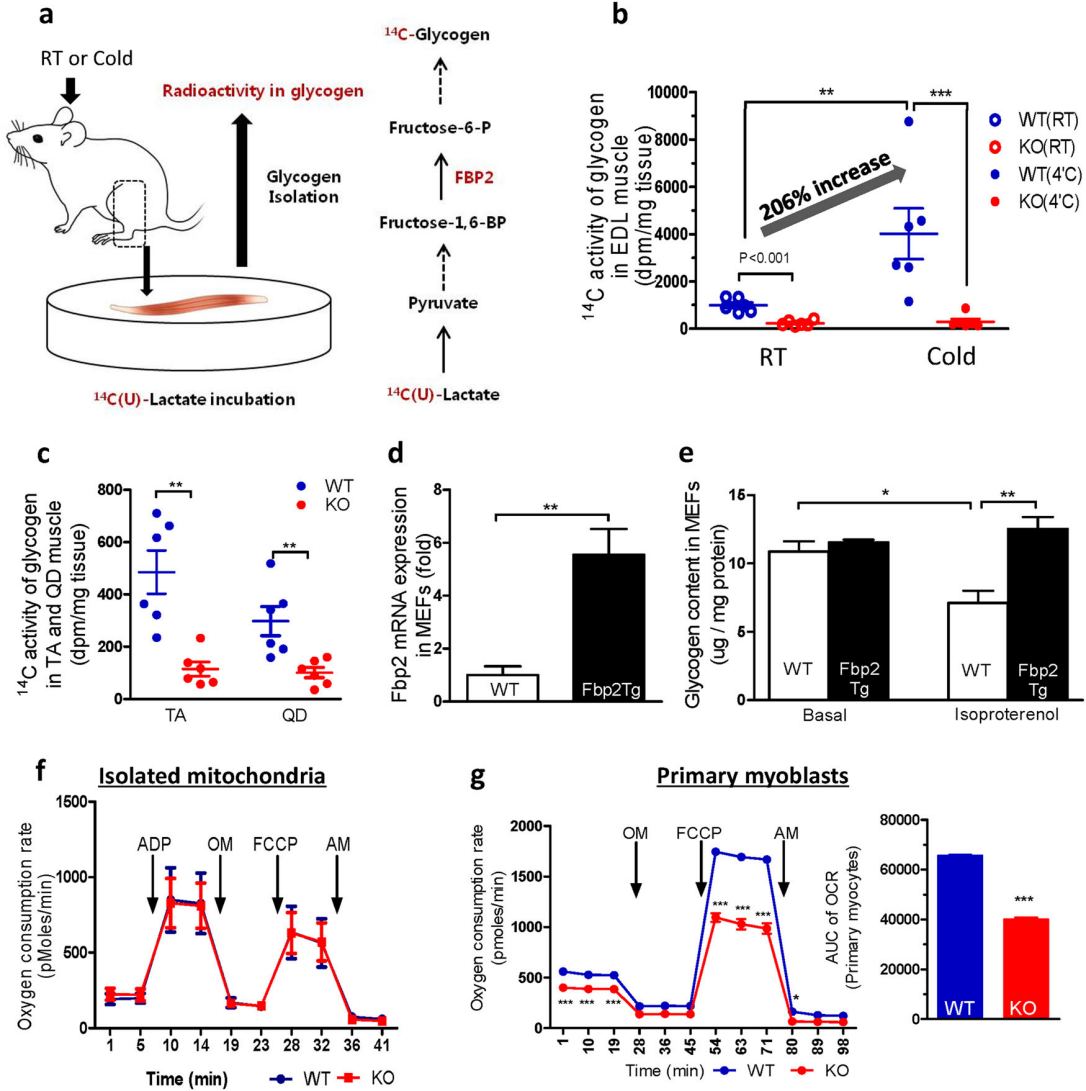
Fbp2 was necessary for intramuscular glyconeogenesis and influenced mitochondrial respiration. **a** Schematic diagram of the ex vivo study of ^14^C(U)-lactate incorporation into glycogen. **b** Results of ^14^C(U)-lactate incorporation into glycogen in the EDL muscles of WT and Fbp2 KO mice under basal conditions and after cold stress, *n* = 6 per group. **c** Results of ^14^C(U)-lactate incorporation into glycogen in the TA and QD muscles of WT and Fbp2 KO mice after cold stress, *n* = 6 per group. **d** Fbp2 gene expression in MEFs isolated from WT and Fbp2 Tg mice analyzed by quantitative RT-PCR, *n* = 6 per group. **e** Glycogen content assay in MEFs isolated from WT and Fbp2 Tg mice under isoproterenol-treated glycogenolytic conditions, *n* = 4 per group. **f** Isolated mitochondrial respiration analysis of WT and Fbp2 KO mice, *n* = 6 per group. **g** Cellular respiration analysis (left) and area under the curve of the oxygen consumption rate (right) of primary myoblasts isolated from WT and Fbp2 KO mice, *n* = 3 per group. EDL extensor digitorum longus, TA tibialis anterior, QD quadriceps, MEFs mouse embryonic fibroblasts, Tg transgenic, ADP adenosine diphosphate, OM oligomycin, FCCP carbonyl cyanide-4-(trifluoromethoxy)phenylhydrazone, AM antimycin. Data are expressed as the mean ± SEM. ^*^P < 0.05, ^**^P < 0.01, ^***^P < 0.001 by one-way ANOVA for (**b**), (**e**) and two-way ANOVA for **f**, (**g**, left). ^**^P < 0.01, ^***^P < 0.001 by Student’s *t*-test for **c**–**d**, (**g**, right).

To address the mechanism underlying the reduced glycogen content in KO mouse muscle, we next tested whether Fbp2 plays a role in intramuscular glyconeogenesis. In the 1960s, it was proposed that a glycogen replenishment pathway other than Cori cycling may exist. A few studies have suggested that intramuscular glyconeogenesis may take part in glycogen replenishment after metabolic stress, such as exercise, and this pathway may be mediated by Fbp2 in skeletal muscle^9,10^; however, this glyconeogenesis has never been tested using Fbp2 KO mice or by challenging with cold stress. Thus, both at room temperature and after 6 h of cold challenge, we conducted experiments using a radioisotope-labeled tracer (^14^C(U)-lactate) to quantitatively measure the incorporation of lactate into glycogen in the skeletal muscle of WT and Fbp2 KO mice (Fig. 4a). Glyconeogenesis was almost completely blocked in the EDL muscle of Fbp2 KO mice, as shown by an ~84% decrease in lactate incorporation into glycogen compared to WT mice at room temperature (Fig. 4b). These results imply that intramuscular glyconeogenesis exists even at room temperature. Since the subthermoneutral temperature of mice is ~30 °C, room temperature (~22 °C) could contribute to the decrease in muscle glycogen content in KO muscle (Fig. 3b). Furthermore, after 6 h of cold challenge, glyconeogenesis was markedly induced by ~206% in the WT mouse muscle, whereas it remained blocked entirely in the muscle of the Fbp2 KO mice (Fig. 4b), implying a more critical role of Fbp2 under cold conditions. Consistent with the EDL muscle results in Fbp2 KO mice, intramuscular glycogen replenishment was significantly decreased in the tibialis anterior (TA) and quadriceps (QD) muscles of Fbp2 KO mice after acute cold challenge compared to WT mouse muscles (Fig. 4c). Furthermore, using mouse embryonic fibroblasts (MEFs) from Fbp2 transgenic mice, we measured the in vitro glycogen content after cold-mimicking stimulation (isoproterenol, a nonselective β-adrenergic receptor agonist). Consequently, the glycogen content was decreased by isoproterenol in WT MEFs but maintained in Fbp2-overexpressing MEFs (Fig. 4d, e), indicating the preservation of glycogen by Fbp2-mediated glyconeogenesis. Furthermore, we investigated whether other heat sources, such as mitochondrial uncoupling and mitochondrial respiratory function, were affected by Fbp2 deletion in WT and KO mice. The cold challenge itself significantly increased the expression of the Pgc-1α gene both in brown adipose tissue and gastrocnemius (GAS) muscle; however, there was no significant difference between genotypes (Supplementary Fig. 3a). After the cold challenge, Ucp3 protein expression was identical between WT and KO mouse muscle, dissociating muscle mitochondrial uncoupling from the cold-intolerant phenotype of KO muscle (Supplementary Fig. 3b, right). The expression of Pgc-1α mRNA (Supplementary Fig. 3a, left) and Ucp1 protein (Supplementary Fig. 3b, left) in brown adipose tissues showed increasing trends in KO mice after cold challenge, which was likely a compensatory response to the decreased body temperature in the Fbp2 KO mice. Accordingly, the expression of mitochondria-related genes in the GAS muscle (Supplementary Fig. 3c) and the mitochondrial oxygen consumption rate with isolated mitochondria from GAS muscle were unchanged (Fig. 4f). However, with primary cultured myoblasts from the WT and Fbp2 KO mouse muscles, cellular oxygen consumption rates were significantly decreased in KO mouse myoblasts (Fig. 4g), which was consistent with the decreased whole-body V_O2_ and V_CO2_ rates in the indirect calorimetry study (Fig. 1h, j). These data suggest that Fbp2 affects thermogenesis mainly by regulating the intracellular glycogen content rather than directly changing mitochondrial function or other thermoregulatory pathways.

## Discussion

The physiological meaning of intramuscular glyconeogenesis has been questioned for over a decade since skeletal muscle mainly replenishes glycogen from exogenous glucose^31,32^ or via liver gluconeogenesis, named Cori cycling^33^. Indeed, when the oxygen supply is insufficient, typically during intensive exercise, lactate produced by anaerobic glycolysis in the muscles moves to the liver and is converted to glucose, which then returns to the muscles^34^. We do not doubt that Cori cycling is the major pathway for preventing lactic acidosis in muscle and further drives additional glycolysis reactions via NAD^+^ generation for most physiological conditions, including fasting and exercise. However, it is not clear whether intramuscular glyconeogenesis is an alternative pathway for replenishing muscle glycogen under specific conditions, such as cold exposure. Furthermore, we questioned why muscle chooses the energetically inefficient method of glycogen replenishment because gluconeogenesis via the liver requires more ATP consumption than glyconeogenesis in skeletal muscle, which bypasses the hexokinase step. In the present study, we discovered that acute cold challenge dramatically induced glyconeogenesis in the EDL muscle of WT mice (Fig. 4b) without increasing the muscle lactate concentration (data not shown). This suggested that muscle glyconeogenesis could have a critical contribution to muscle glycogen replenishment after cold challenge, which might mimic aerobic glycogenolytic conditions.

Moreover, in the present study, we found the physiological importance of Fbp2 in the thermal homeostasis of the whole-body under acute cold stress and demonstrated intramuscular glyconeogenesis as an underlying mechanism of thermogenesis. These findings support an old hypothesis by Holloszy in 1979^9^, which stated that the existence of intramuscular glyconeogenesis and the involvement of Fbp2 in this pathway might contribute to glycogen replenishment from endogenous carbon sources. However, whether lactate can be resynthesized to carbohydrates in skeletal muscle under physiological conditions is controversial, and the physiological role of Fbp2 has been unclear for two decades^35–37^. This is likely due to the following three reasons. First, the majority of previous studies focused on exercise conditions^9,10,38^, where most lactate was oxidized by the mitochondria rather than converted to glycogen due to the increased ATP demand after physical activity. Second, previous studies have focused on glycogen synthesis with exogenous glucose supply^31,32^. Therefore, it might be difficult to investigate the role of intramuscular glyconeogenesis in skeletal muscle glycogen metabolism. Third, experimental evidence supporting this concept^9,10,38,39^ has never been conducted under the in vivo condition of Fbp2 deficiency. We believe that the most important experimental design of our study that differentiated it from previous studies was the cold challenge without exogenous glucose supply. First, in the present study, we found that Fbp2 KO mice exhibited decreased whole-body energy metabolism only during the light phase when food intake was minimized. Second, we used the hyperinsulinemic-euglycemic clamp technique to mimic the feeding state, which demonstrated that there was no difference in exogenous glucose uptake between Fbp2 WT and KO mice. Third, under fasting conditions, Fbp2 KO mice were cold intolerant and severely hypothermic after the acute cold challenge. However, when food was supplied, the Fbp2 KO mice survived well without any decreases in body temperature even after 3 weeks of the cold challenge. Consequently, these results indicate that intramuscular glyconeogenic flux is crucial for the maintenance of the endogenous glycogen pool and muscle thermogenesis.

However, our study also had certain limitations and assumptions that were not fully addressed. First, we assumed that the fructose futile cycling rate would be affected by the decreased glyconeogenesis in the Fbp2 KO mice, but we could not provide any in vivo flux data for the futile cycling rate in the Fbp2 KO muscle due to the technical limitations of the tracer methods. Future studies with a new isotope tracer methodology may provide more direct evidence for futile thermogenesis. Second, there are further questions that need to be addressed in future studies regarding whether the present findings of this study can be extended to clinical interpretation in humans. Although we focused on the relationship between cold tolerance and intramuscular glyconeogenesis, the physiological role of Fbp2 may be extended to other conditions where skeletal muscle glycogen breakdown replenishment is increased. We demonstrated in the present study that Fbp2 is closely related to the endogenous glucose source of skeletal muscle; therefore, further experiments with aerobic exercise or prolonged fasting could provide clinical insight into Fbp2 in various physiological conditions.

In summary, the present study highlights new knowledge regarding Fbp2 in glycogen metabolism and provides information on the importance of intramuscular glyconeogenesis on energy balance and thermal homeostasis in vivo. Furthermore, the present study suggests that Fbp2-mediated intramuscular glyconeogenesis is a novel mechanism contributing to muscle glycogen replenishment and thermogenesis when exogenous glucose supplementation is limited.

## Supplementary information

Supplementary information
