# Supplementary material for: The essential role of fructose-1,6-bisphosphatase 2 enzyme in thermal homeostasis upon cold stress
Source: Exp Mol Med. 2020 Mar 16;52(3):485–96. doi: 10.1038/s12276-020-0402-4 (PMC7156669; doi:10.1038/s12276-020-0402-4)
Supplement: Supplementary file 1 — Supplementary information [file 12276_2020_402_MOESM1_ESM.docx]

**Supplementary Information**

**
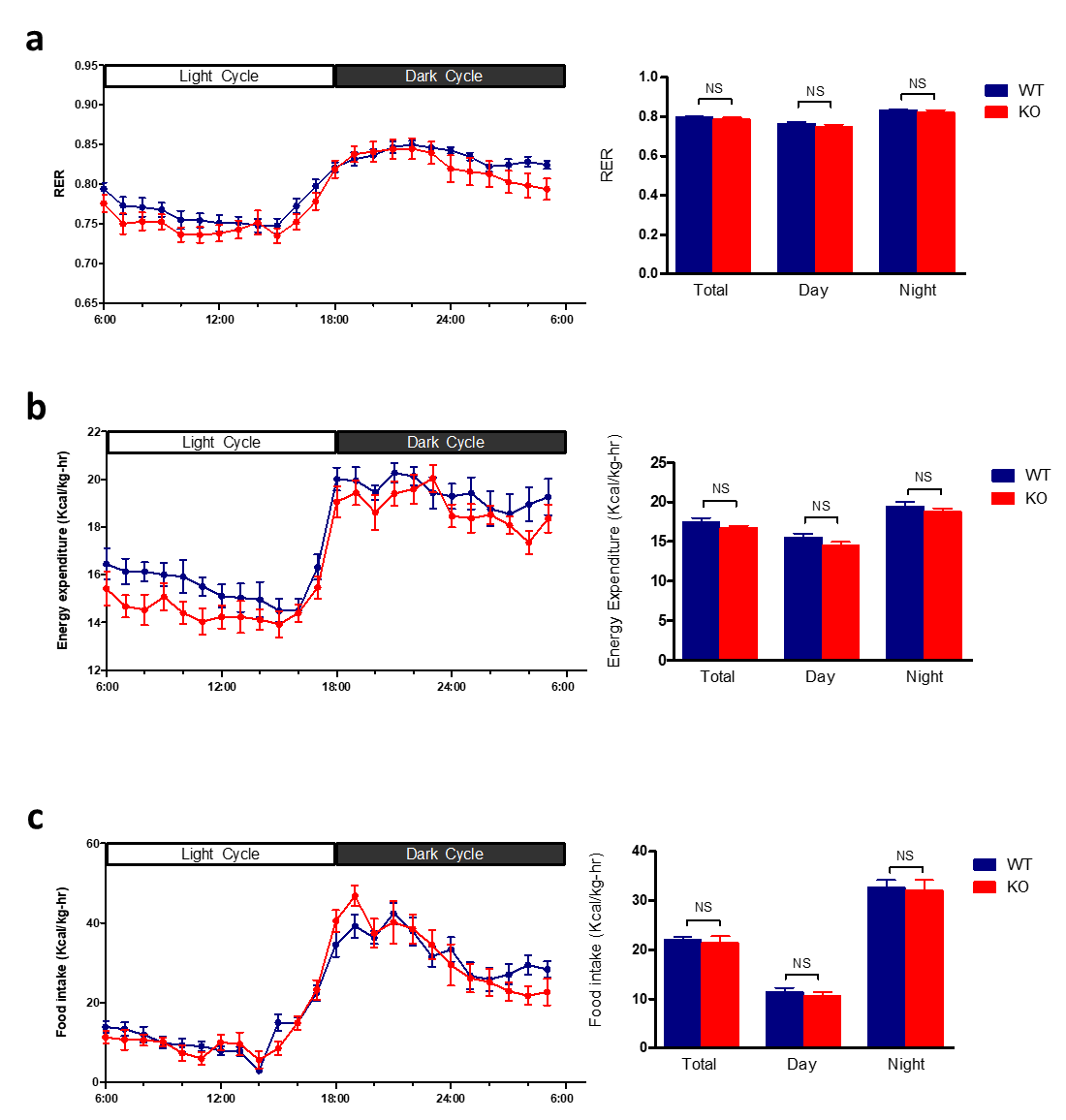
**

**Supplementary Figure 1. Energy metabolism of WT and KO mice using the comprehensive lab animal monitoring system (CLAMS).** Time course average of each time points (left) and average of day and night time (right) during the CLAMS analysis. (a) Respiratory exchange ratio (RER), (b) Energy expenditure, (c) Food intake of WT and Fbp2 KO mice during 72hrs analysis. n=8 each. Data are expressed as mean ± SEM analyzed by Two-Way ANOVA for (left) and Student’s t-test for (right). NS: Non-significant.


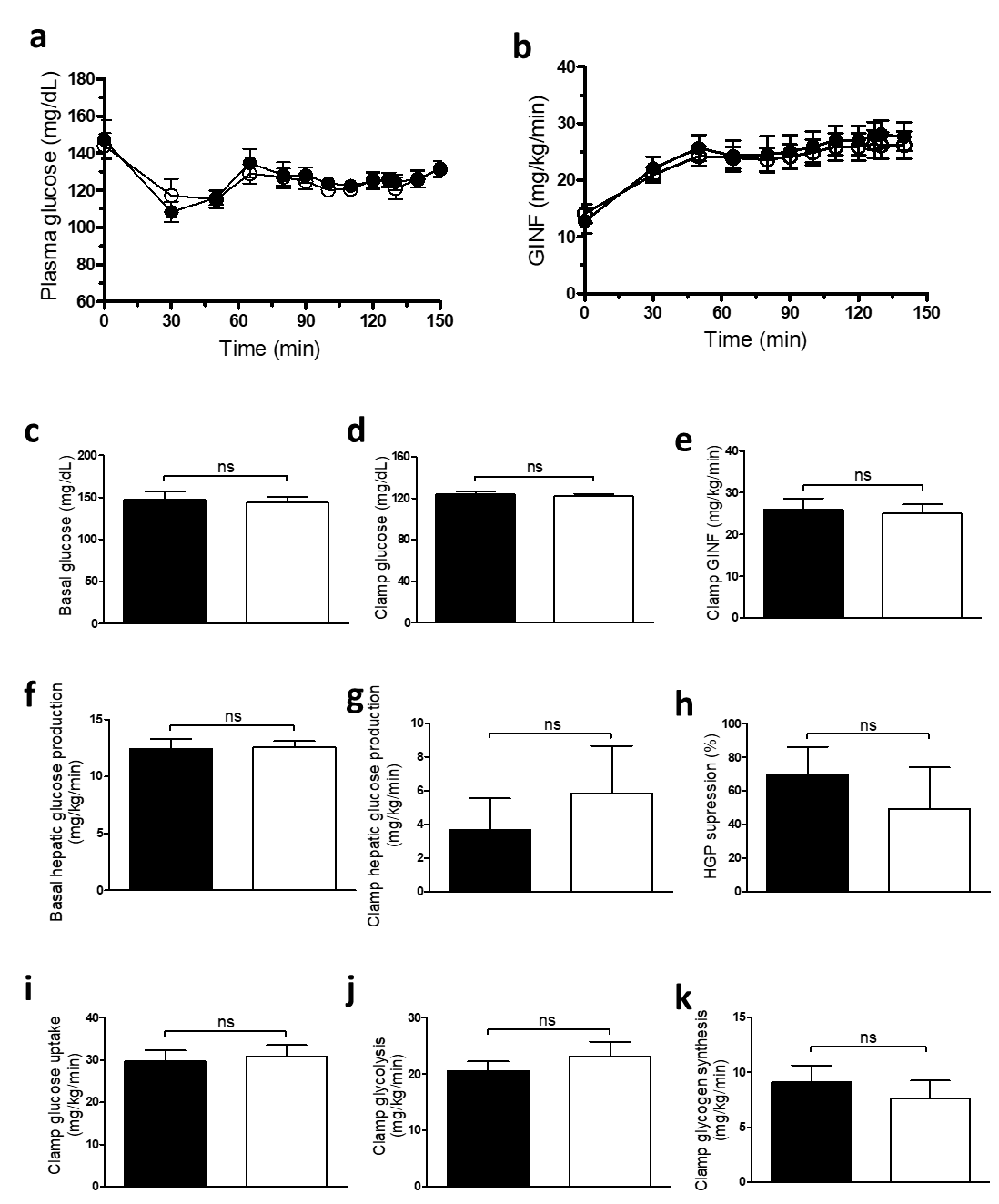


**Supplementary Figure 2. In vivo glucose metabolism measured by hyperinsulinemic-euglycemic clamp in WT and Fbp2 KO mice after high fat diet feeding.** Time course plots of (a) plasma glucose concentration and (b) glucose infusion rate during the hyperinsulinemic-euglycemic clamp study. (c) Basal glucose, (d) clamped (average of 120-140 min) glucose concentration, (e) clamped glucose infusion rate, (f) basal hepatic glucose output rate, (g) clamped hepatic glucose output rate, (h) the percentage of insulin-suppressed hepatic glucose output, (i) clamped glucose uptake rate, (j) clamped glycolysis rate, (k) clamped glycogen synthesis rate during the hyperinsulinemic-euglycemic clamp study. WT: n=8, Fbp2 KO: n=9. Data are expressed as mean ± SEM and analyzed by Two-Way ANOVA with post-hoc analysis for (a), (b) and by Student’s t-test for (c-k). NS: Non-significant.

**
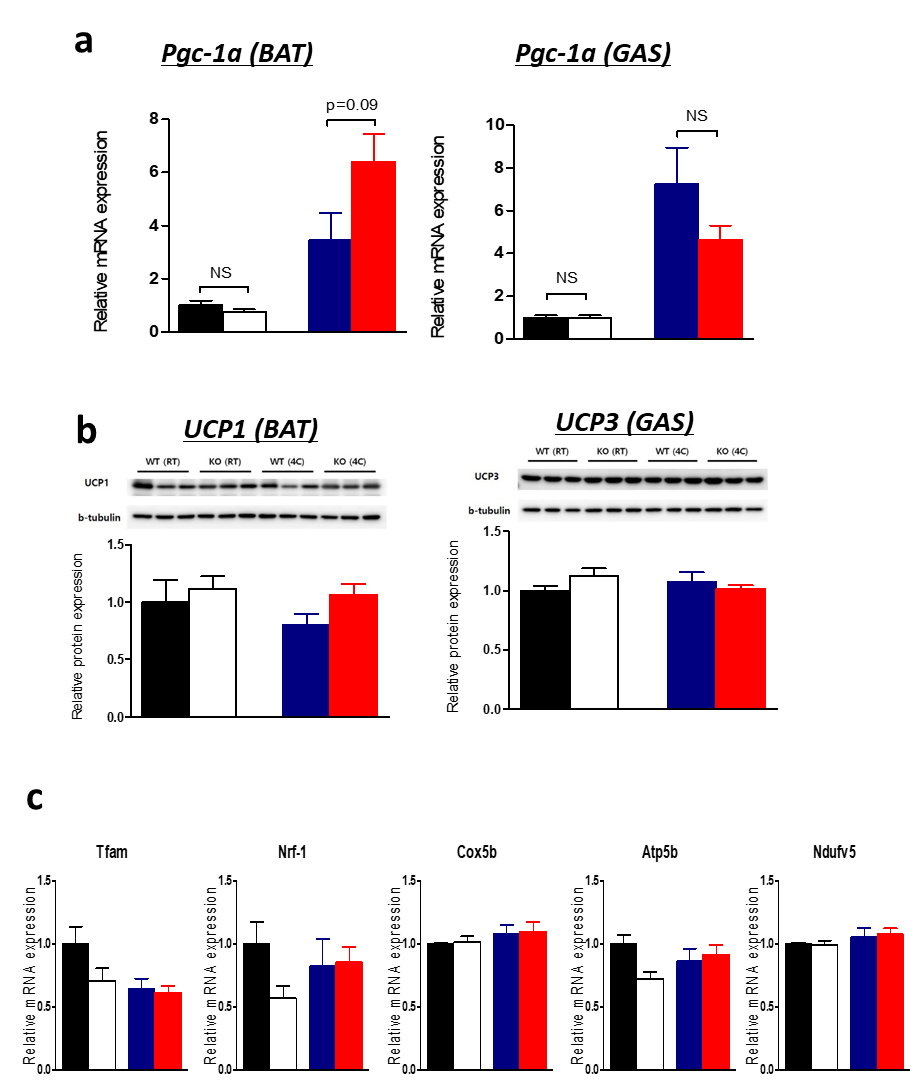
**

**Supplementary Figure 3. Mitochondrial uncoupling and mitochondrial-related gene expressions in various tissues from WT and Fbp2 KO mice.** (a) Pgc-1α gene expression patterns in brown adipose tissue (BAT) and GAS muscle of WT and Fbp2 KO mice in cold stress experiments. WT: n=4 each, KO: n=6 each. (b) UCP1 protein expression in BAT and UCP3 protein expression in GAS of WT and Fbp2 KO mice in cold stress experiments. WT: n=4 each, KO: n=6 each. (c) Mitochondrial gene expression analysis in GAS of WT and Fbp2 KO mice. Mitochondrial transcription factor A (Tfam), Nuclear respiratory factor 1 (Nrf-1), Cytochrome c oxidase subunit 5B (Cox5b), ATP synthase F1 subunit beta (Atp5b) and NADH dehydrogenase [ubiquinone] flavoprotein 5 (Ndufv5). WT: n=4, KO: n=6. Data are expressed as mean ± SEM and analyzed by One-Way ANOVA with post-hoc analysis. NS: Non-significant.
